# Interplay of the red blood cell and vascular endothelial nitric oxide synthase system to combat cardiac complications of anemia

**DOI:** 10.1007/s00395-020-0801-7

**Published:** 2020-06-12

**Authors:** Andreas Daiber, Thomas Münzel

**Affiliations:** 1 Center for Cardiology, Cardiology I, University Medical Center Mainz, Geb. 605, Langenbeckstr. 1, 55131 Mainz, Germany; 2 German Center for Cardiovascular Research (DZHK), Partner Site Rhine-Main, Mainz, Germany

## The cardiovascular protective profile of endothelial nitric oxide synthase (eNOS) and nitric oxide (^·^NO)

The mechanism of cardioprotection described in the clinically important study by Wischmann et al. is based on the assumption of a compensatory enhancement of vascular endothelial nitric oxide synthase (eNOS) activity and nitric oxide (^·^NO) formation in anemic mice, when red blood cell (RBC) eNOS function and ^·^NO formation are impaired [28]. The cardioprotective properties of eNOS and ^·^NO are widely accepted and were extensively reviewed in the past [9, 24] and redefined in recent years [3]. The cardioprotection afforded by ^·^NO (e.g., from nitrovasodilators such as nitroglycerin [13]) largely depends on the prevention of mitochondrial permeability transition pore (mPTP) opening via S-nitros(yl)ation of the mPTP regulator cyclophilin D during reperfusion [4]. This mechanism reflects a major detrimental process in ischemia/reperfusion (I/R) damage leading to excessive reactive oxygen species (ROS) formation/release as well as onset of apoptotic cell death [7, 21]. Oxidative stress in general plays an important role for development and progression of cardiovascular diseases [19], especially for I/R associated events such as myocardial infarction [9]. Importantly, ^·^NO can directly reduce I/R-dependent ROS formation by suppression of mitochondrial respiratory complex I activity via S-nitros(yl)ation [5]. Nitric oxide is also implicated in pre-, post- and remote-conditioning, drug- and non-drug-based therapeutic concepts currently discussed for cardioprotection [2, 14, 23].

**Fig. 1:**
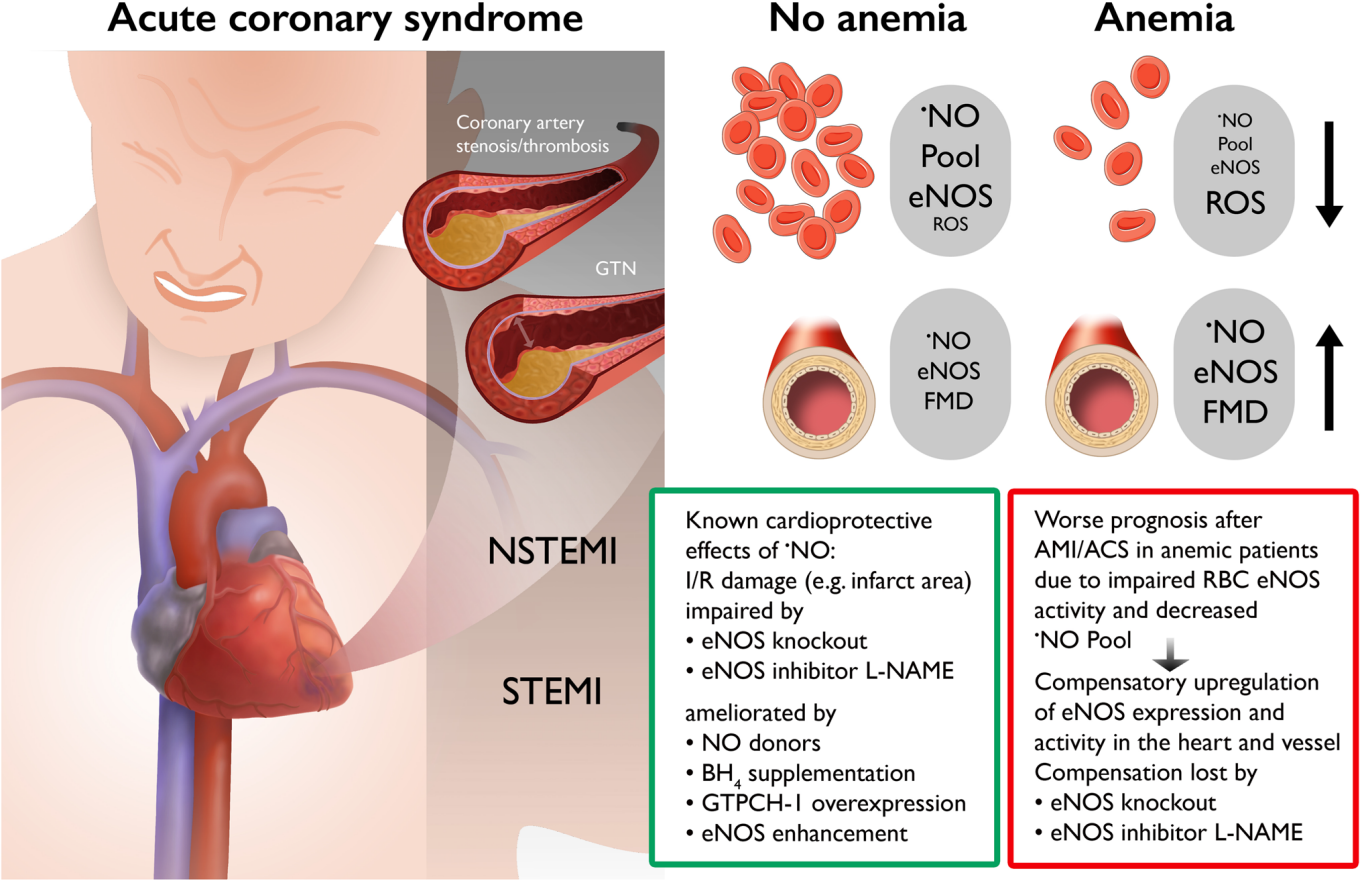
Scheme illustrating the mechanisms of cardioprotection by nitric oxide or the aggravation of ischemic cardiac damage by impaired nitric oxide signaling. Nitric oxide confers known cardioprotective effects and attenuates I/R cardiac damage (as observed in STEMI and NSTEMI [17]). The protective effects are lost by genetic or pharmacological eNOS inhibition. Implications for the novel findings on interplay of red blood cell/vascular eNOS for cardioprotection in a model of anemia are described [28]. In principle, anemic patients have a worse prognosis after AMI or ACS due to dysfunctional RBC ^·^NO signaling, but upregulation of eNOS activity in the heart and vessels of anemic patients provides compensatory protection that is lost upon genetic or pharmacological eNOS inhibition. *GTPCH-1* GTP-cyclohydrolase-1, *GTN* glycerol trinitrate (nitroglycerin), *STEMI* ST-elevation myocardial infarction, *NSTEMI* non-STEMI. Contains images from Servier Medical Art by Servier, licensed under a Creative Commons Attribution 3.0 Unported License

The general role of ^·^NO for cardioprotection is also supported by numerous reports on loss of cardioprotective effects of ^·^NO or ^·^NO-related therapies upon treatment with the inhibitor of all NOS isoforms, *N*^G^-nitro-l-arginine methyl ester (l-NAME) (only citing a few [4, 6, 15, 26]). Also exogenous administration of tetrahydrobiopterin (BH_4_), an essential cofactor for eNOS function, improved ischemic damage in isolated hearts subjected to I/R [27, 29]. Likewise, cardiac-specific overexpression of GTP-cyclohydrolase-1, the rate-limiting enzyme for tetrahydrobiopterin synthesis, improved ischemic preconditioning [12] and also attenuated post-infarction cardiac remodeling [16], most probably by restoration of tetrahydrobiopterin synthesis and thus by the prevention of eNOS uncoupling [10]. Further support of this concept is provided by genetic models, where eNOS knockout mice showed more pronounced ischemic damage, myocardial fibrosis and impaired left-ventricular end-diastolic volume and ejection fraction, when subjected to myocardial infarction [25]. Of note, genetic deficiency in neuronal nitric oxide synthase (nNOS) or inducible nitric oxide synthase (iNOS) did not show this aggravated ischemic damage; in contrast, iNOS knockout mice were rather protected against ischemic damage [25]. Also, the cardioprotective effects of nitroglycerin upon myocardial infarction were lost in eNOS knockout mice [4]. In addition, a cardiomyocyte-specific overexpression of eNOS largely prevented I/R injury [8]. The proof for the central role of eNOS-derived ^·^NO to prevent or at least attenuate ischemic heart damage was based on decreased infarct size and cardiac oxidative stress upon coronary artery ligation by therapy with the eNOS enhancer AVE9488, whereas these protective effects were virtually absent in eNOS knockout mice [11]. All these different regulators of eNOS activity, ^·^NO formation and endothelial function are summarized in the Fig. 1 and have been-reviewed previously [18, 22], and put into context with the novel findings by Wischmann et al. [28].

## Essential interplay of red blood cell and vascular eNOS for nitric oxide-mediated cardioprotection against I/R injury in anemia

With the present studies, Wischmann et al. show that cardiovascular protection against I/R damage in response to acute myocardial infarction (AMI) is mainly based on endogenous ^·^NO formation from either red blood cell (RBC, e.g., by nitrite bioactivation or RBC-eNOS) or vascular/cardiac eNOS [28]. In a mouse model of anemia, the authors demonstrate that AMI-induced mortality is more pronounced due to impaired RBC-derived ^·^NO formation as also seen with the increase in AMI mortality upon pharmacological eNOS inhibition by l-NAME. The most deleterious outcome was observed when anemic mice were treated with l-NAME and then subjected to AMI. The demonstration that AMI did not induce a severe impairment of cardiac functional parameters in the setting of anemia was attributed to the finding that cardiac and vascular eNOS were upregulated in the anemic mice—this was suggested by the authors as a compensatory mechanism. The impaired circulating ^·^NO pool in anemic mice could be explained mainly by impaired RBC-derived ^·^NO formation due to RBC dysfunction as indicated by lower RBC hemoglobin and iron content, whereas cell-free hemoglobin was increased and RBC redox state was impaired (e.g., higher ROS levels and lower reduced glutathione levels). As a proof of concept, the authors show that transfer of RBC from anemic or eNOS knockout mice to wild type mouse hearts subjected to I/R prevented the recovery of cardiac function as compared to the wild type mouse hearts, when healthy wild type RBC were present. This proof-of-concept experiment was also confirmed using RBC from patients with acute coronary syndrome (ACS) with and without anemia. The RBC from ACS patients with anemia caused a significantly worse recovery in wild type mouse hearts that were subjected to I/R as compared to RBC from ACS patients without anemia, further substantiating the proposed concept that RBC-derived ^·^NO plays a major role for cardioprotection against I/R damage. In line with these observations, patients with chronic severe anemia had substantially increased forearm blood flow (measured by plethysmography) and showed more pronounced decrease in forearm blood flow upon l-NAME administration as compared to  healthy subjects [1], supporting the postulated compensatory activation of vascular eNOS in the state of dysfunctional RBC eNOS.

## Implications of eNOS function and nitric oxide bioavailability in patients with ACS and anemia

Thus, in summary, the results of this highly important study demonstrate that moderate blood loss anemia is associated with severe red blood cell dysfunction and increased superoxide production, which may be related at least in part to eNOS uncoupling in RBC leading to a reduction of the ^·^NO pool. In addition, Wischmann et al. also established with a series of well-designed experiments for the first time that vascular and cardiac eNOS are crucial for the cardiocirculatory adaptation to anemia in particular in the setting of I/R [28]. The presented findings will help to improve therapeutic strategies in the setting of AMI and anemia. Thus, the principle target will not be solely the reduced hemoglobin level, but also the normalization of the reduced ^·^NO pool in erythrocytes or the enhancement of eNOS activity in the vasculature and the myocardium. This should also be considered for blood transfusion since the duration of storage of RBC in the transfusion department is negatively correlated with flow-mediated dilation measured in anemic subjects after transfusion, indicating that RBC ^·^NO formation capacity is impaired upon prolonged storage [20]. It remains to be established, whether nitric oxide donor therapy, tetrahydrobiopterin treatment or eNOS enhancement will decrease the cardiovascular risk of patients with anemia.
